# Parents as Agents of Change (PAC) in pediatric weight management: The protocol for the PAC randomized clinical trial

**DOI:** 10.1186/1471-2431-12-114

**Published:** 2012-08-06

**Authors:** Geoff D C Ball, Kathryn A Ambler, Rachel A Keaschuk, Rhonda J Rosychuk, Nicholas L Holt, John C Spence, Mary M Jetha, Arya M Sharma, Amanda S Newton

**Affiliations:** 1 8B, Pediatric Centre for Weight and Health, Edmonton General Continuing Care Centre, 11111 Jasper Ave, Edmonton, AB, CANADA, T5K0L4; 2 8B, Pediatric Centre for Weight and Health, Edmonton General Continuing Care Centre, 11111 Jasper Ave, Edmonton, AB, CANADA, T5K0L4; 3 Community Programs East, Alberta Health Services, Northgate Health Centre, Edmonton, AB, CANADA; 4 Edmonton Clinic Health Academy, Department of Pediatrics, University of Alberta, Rm 3-524, 11405 87 Avenue, Edmonton, AB, CANADA, T6G1C9; 5 P3-20 S Van Vliet Centre, Faculty of Physical Education and Recreation, University of Alberta, Edmonton, AB, CANADA, T6G2H9; 6 W1-16 H Van Vliet Centre, Faculty of Physical Education and Recreation, University of Alberta, Edmonton, AB, CANADA, T6G2H9; 7 8B, Pediatric Centre for Weight and Health, Edmonton General Continuing Care Centre, 11111 Jasper Ave, Edmonton, AB, CANADA, T5K0L4; 8 406 CSC Royal Alexandra Hospital, Edmonton, AB, CANADA, T5H3V9; 9 3-526 Edmonton Clinic Health Academy, Department of Pediatrics, University of Alberta, 11405 87 Avenue, Edmonton, AB, CANADA, T6G1C9

**Keywords:** Obesity, Pediatric, Treatment, Parents, Cognitive behavioral therapy, Canada

## Abstract

**Background:**

There is an urgent need to develop and evaluate weight management interventions to address childhood obesity. Recent research suggests that interventions designed for parents exclusively, which have been named *parents as agents of change* (PAC) approaches, have yielded positive outcomes for managing pediatric obesity. To date, no research has combined a PAC intervention approach with cognitive behavioural therapy (CBT) to examine whether these combined elements enhance intervention effectiveness. This paper describes the protocol our team is using to examine two PAC-based interventions for pediatric weight management. We hypothesize that children with obesity whose parents complete a CBT-based PAC intervention will achieve greater reductions in adiposity and improvements in cardiometabolic risk factors, lifestyle behaviours, and psychosocial outcomes than children whose parents complete a psycho-education-based PAC intervention (PEP).

**Methods/Design:**

This study is a pragmatic, two-armed, parallel, single-blinded, superiority, randomized clinical trial. The primary objective is to examine the differential effects of a CBT-based PAC *vs* PEP-based PAC intervention on children’s BMI z-score (primary outcome). Secondary objectives are to assess intervention-mediated changes in cardiometabolic, lifestyle, and psychosocial variables in children and parents. Both interventions are similar in frequency of contact, session duration, group facilitation, lifestyle behaviour goals, and educational content. However, the interventions differ insofar as the CBT-based intervention incorporates theory-based concepts to help parents link their thoughts, feelings, and behaviours; these cognitive activities are enabled by group leaders who possess formal training in CBT. Mothers and fathers of children (8–12 years of age; BMI ≥85^th^ percentile) are eligible to participate if they are proficient in English (written and spoken) and agree for at least one parent to attend group-based sessions on a weekly basis. Anthropometry, cardiometabolic risk factors, lifestyle behaviours, and psychosocial health of children and parents are assessed at pre-intervention, post-intervention, 6-, and 12-months follow-up.

**Discussion:**

This study is designed to extend findings from earlier efficacy studies and provide data on the effect of a CBT-based PAC intervention for managing pediatric obesity in a real-world*,* outpatient clinical setting.

**Trial Registration:**

ClinicalTrials.gov identifier: NCT01267097

## Background

Pediatric obesity has increased dramatically in economically developed countries over the past several decades [1]. The most recent national estimates from the Canadian Community Health Survey revealed that 26% of Canadian children and youth were overweight while 8% were obese [2]. These data showed overweight and obesity increased 50% and 167%, respectively, since measured height and weight data were last collected from Canadian children in the late 1970s. This is an alarming trend since a high level of body fat in children and youth is linked to numerous adverse health outcomes including high blood pressure, dyslipidemia, insulin resistance, and non-alcoholic fatty liver disease [3]. In addition to the high proportion of boys and girls impacted by obesity-related medical co-morbidities, the psychosocial consequences of having an unhealthy weight are likely most salient for families. A number of reports have studied the connections between pediatric obesity and depression [4,5], anxiety [5], self-esteem [6], body image [7], executive functioning [8], as well as bias and stigmatization [9,10]. Collectively, this body of evidence highlights the variety of factors that influence the health and well-being of children with obesity.

There have been numerous calls for the development and evaluation of pediatric weight management interventions [11-13]. Such interventions are usually designed to improve lifestyle behaviours to reduce adiposity and risk factors for chronic diseases including type 2 diabetes and cardiovascular disease. Though researchers outside of Canada have established fundamental features of pediatric weight management, their application in a Canadian context has been limited. The Canadian Obesity Clinical Practice Guidelines (CPG) reinforced this knowledge gap [11]; notably, of all the weight management research used to inform the CPG, none of the evidence was Canadian. This situation is undesirable since Canada’s cultural, social, geographic, and health services uniqueness suggests that weight management interventions that work in other countries may not translate universally or successfully [14,15]. Despite these issues, a common theme underscored in almost every published report on pediatric weight management is the central role played by parents [16,17].

Parents play an invaluable role in creating a supportive home environment to enable their children to make healthy lifestyle choices [18,19]. Parents also serve as important role models given that parental attitudes and behaviours regarding physical activity and nutrition can have a substantial impact, both positively and negatively, on the attitudes and behaviours of their children [20-24]. Parenting style is also an important factor that impacts child health outcomes. In research extending Baumrind’s classical descriptions of parenting styles [25], children of parents who demonstrate controlling, restrictive behaviours exhibit less healthy dietary behaviours and are at increased risk of obesity *versus* children of parents who demonstrate more supportive, authoritative practices [26-30]. In addition, data on psychosocial stress within families suggests that improving the family system and parent–child relationships may reduce the risk of pediatric obesity [31]. Taken together, these observations support the need for weight management interventions that attend to both cognitive and behavioural factors within the family context.

The key role played by parents in pediatric weight management interventions was established in the early 1980s by the formative research conducted by Leonard Epstein and colleagues [28,32-35]. Their work has been extended in recent years through research focused on parents as agents of change (PAC) in lieu of treating parents and children as a dyad. Intervening with parents exclusively to address other health concerns in children and youth has been applied successfully in the past [36-38], but represents a newer model for care for pediatric weight management. If parent-only interventions are as effective as interventions that include children only or parent–child dyads, a parent-only model would be the most efficient (and likely cost-effective) treatment option. Indeed, emerging evidence supports a PAC treatment approach. For instance, in a randomized, one-year study of obese 6 – 11 year olds, [Golan et al. 39] compared two weight management interventions (one for children exclusively *versus* one for parents exclusively). While children in both groups lost weight, the reduction in percent overweight was greater for children in the parent-only group compared to those in the group that included children exclusively; program adherence and retention were also superior in the parent-only group. More recently, Golan and colleagues compared child weight loss in parent-only *versus* parent + child groups. Obese children of parents in the parent-only group showed greater reductions in adiposity compared to children in the parent + child group, improvements that were maintained at 18-months follow-up [40]. Since these initial reports, data from larger, higher-quality clinical trials have confirmed the beneficial effects of PAC interventions in pediatric weight management [41-44].

Though the aforementioned data have highlighted the fundamental leadership role parents can play in helping their children to achieve success in pediatric weight management, the interventions have provided limited insight into how and why the interventions promote behavioural and cognitive changes [45]. The current study builds on the seminal work of Golan and colleagues by applying a PAC approach, which includes a theoretically-based, clinical treatment modality (cognitive behavioural therapy, CBT) in comparison to the traditional psycho-educational approach that helps to interpret study outcomes. Although CBT has been used previously for treating pediatric obesity [46,47], our study addresses family-oriented issues with parents exclusively rather than focusing on boys and girls themselves. We believe that working with parents on their own may also allay concerns regarding intervening with children (i.e., stigmatization, low motivation, a lack of identifying obesity as a health concern). Further, by evaluating a PAC CBT-based intervention in an out-patient clinical setting in our local children’s hospital, the current trial is designed to expand on the promising findings from efficacy-based studies to determine the effectiveness of PAC interventions in a real-world environment, which often includes children with severe obesity. This paper describes the protocol our team is using to examine two PAC-based interventions for pediatric weight management. We hypothesize that children whose parents complete the CBT-based PAC intervention will achieve greater reductions in adiposity, improved lifestyle behaviours and psychosocial outcomes, and decreased cardiometabolic risk factors compared to children whose parents complete a psycho-education-based (PEP) PAC intervention, which is similar in content and structure to the CBT-based version, but does not include elements of cognitive behavioural therapy.

## Methods/Design

This study is a pragmatic, two-arm, parallel, single-blinded, superiority randomized clinical trial. The primary objective is to examine the differential effects of CBT-based PAC *vs* PEP-based PAC on children’s BMI z-score (primary outcome). Secondary objectives are to examine intervention-mediated changes in metabolic, behavioural, and psychosocial variables in children and parents. The RCT is conducted at the Pediatric Centre for Weight and Health (PCWH), which is an outpatient pediatric weight management clinic at the Stollery Children’s Hospital (Alberta Health Services) in Edmonton, Alberta, Canada. As a clinical program that offers weight management care to boys and girls with obesity (and their families), we have used different study designs to evaluate weight management interventions in the past, which has included applying a wait-list control group [48]. However, based on our team’s collective clinical and research experience, a study design that includes offering health services to families as close as possible to their entry into our clinic satisfies our institutional commitment to provide timely access to weight management health services. With this in mind, developing and comparing two PAC interventions (CBT and PEP) that are similar but distinct meets our academic aim to compare the effectiveness of two evidence-informed approaches [49].

### Ethical considerations

The PAC RCT is registered with ClinicalTrials.gov (NCT01267097) and has received approval from both the Human Research Ethics Board (University of Alberta) and the Northern Alberta Clinical Trials and Research Centre. Family recruitment is conducted by a research coordinator within our weight management clinic. Informed and written consent and assent are obtained from parents and children, respectively, at the point of recruitment. Families who are referred to our weight management clinic and satisfy our inclusion criteria, but decline participation, still receive weight management health services, so no families are refused care. In recognition of families’ time and effort, tokens of appreciation (i.e., gift cards to a local shopping centre) are offered to families at each data collection time point (pre-intervention, post-intervention, 6- and 12-months follow-up).

### Recruitment

Families are eligible for this study if (*i*) children are between 8 – 12 years old, (*ii*) children present with an age- and sex-specific BMI ≥85^th^ percentile [50], (*iii*) at least one parent agrees to attend weekly PAC sessions for 16 weeks, and (*iv*) children and parents are fluent in English (verbal and written). Recruitment is conducted primarily through the PCWH, although we also attempt to recruit families from the greater Edmonton area through advertisements (e.g., posters on local health, community, and recreation centers, mail outs to local family and pediatric medical clinics).

### Timeframe

Data are collected from families at pre-intervention, post-intervention, 6-, and 12-months follow-up. For practical reasons, at each time point, data collection occurs within a 2-month window (e.g., within 8 weeks before the first PAC session, within 8 weeks after the last PAC session, and between 4 weeks before to 4 weeks after each of the 6- and 12-month follow-up time points). Clinical and research experience by our team [48] revealed that this timeframe is needed to accommodate families’ schedules and clinical capacity to complete data collection, which also differentiates our real-world, comparative effectiveness research [51] from more stringent criteria that are applied in the context of efficacy studies. Data collection is completed by clinicians working within the PCWH, and given the real-world, applied health services environment within which the study is conducted, we are unable to blind them from knowing which families are receiving the CBT- and PEP-based versions of the PAC intervention [52].

### The PAC interventions: design and delivery

The PAC curriculum (please see Table 1) was developed using strategies consistent with intervention mapping [53] by incorporating evidence-based, lifestyle and behavioural strategies for pediatric weight management [54]. Both the CBT and PEP interventions consist of 16 weekly sessions that are offered to parents (in the evening) twice yearly (September – December; January – April). Sessions are held within our health care institution in classrooms within different areas of the hospital as a means to minimize cross-group contamination. Two healthcare professionals (e.g., dietitian, nurse, fitness professional, mental health professional) per intervention arm provide the curriculum to small groups of parents that will range in size to no more than 15 parents/group. Although at least one parent per family is required to attend the group sessions, additional parents or caregivers are welcome.

**Table 1:** An overview of the Parents as Agents of Change (PAC) intervention curriculum

**Session**	**Title**	**Content overview**
1	*Welcome*	Introduction and orientation; parents receive pre-intervention snapshot of their children’s lifestyle, behavioural and metabolic health measurements, which is used throughout the intervention to inform decisions regarding behaviour change priorities and goal-setting
2	*Food For Thought*	Canada’s Food Guide, PAC nutrition goal #1 (intake of vegetables and fruit)
3	*More Food For Thought*	Canada’s Food Guide, PAC nutrition goals #2 (intake of whole grain products) and #3 (intake of sugar-sweetened beverages)
4	*It’s Your Move*	Canada’s Physical Activity Guide for Children, PAC physical activity goal #1 (steps / day)
5	*Screensavers*	Canada’s Physical Activity Guide for Children, PAC physical activity goal #2 (leisure time screen time)
6	*Portion Distortion*	Food labels, serving sizes *vs* portion sizes
7	*Move It!*	Canada’s Physical Activity Guide for Children, PAC physical activity goal #3 (moderate-to-vigorous physical activity)
8	*Staying On Track*	Behaviour change relapse prevention
9	*Get A Cue!*	Social cues influencing nutrition and physical activity
10	*Positive Parenting Partnerships*	Limit-setting, boundaries, communication
11	*Community Connections*	Environmental factors influencing nutrition, physical activity
12	*Eating Out, Eating Healthy*	Menu reading, providing healthy lunches
13	*Feelin’ Groovy!*	Healthy self-esteem and body image
14	*Time Management*	Family-based priority setting
15	*He Said, She Said*	Bullying, sibling rivalry, peer relationships
16	*Ready, Set, Go!*	Intervention review, future planning and goal-setting

The PAC intervention includes a variety of different educational approaches such as didactic teaching, individual/private parent activities, group-based brain-storming/problem-solving, and inter-active experiential learning. The intervention content is presented to parents through a *PowerPoint*® slide set; as group leaders deliver the manualized content, parents follow along with their individual parent manuals (please see Additional file 1: Appendix for sample content from both leader and parent manuals). Leader manuals include the individual slides, information to be presented to the group, description of the aforementioned learning activities for parents, and a list of references (i.e., journal articles, books) that were used to develop the curriculum. Parent manuals include a copy of the slides, descriptions of the intervention activities, and lined spaces for parents to record notes and complete goal-setting activities on a week-by-week basis. Additional information (i.e., educational handouts, local resources) is made available for parents within the classroom on a community resource table. In lieu of potentially overloading parents with information related to nutrition, physical activity, behaviour change, and local resources for families, a decision was made by our team during the intervention development phase to limit the curriculum to what we viewed as ‘need to know’ information that was directly related to pediatric weight management. Information on topics that are team considered was ‘nice to know’ was removed from the curriculum and provided to families on the community resource table, which is intended to provide parents with optional information that parents may find interesting and relevant, but is not central to pediatric weight management and integral to helping families make healthy behaviour changes.

### The PAC intervention: similarities and differences between CBT and PEP versions

Both intervention arms in the trial are the same in frequency of contact (16 sessions), content (identical information is delivered), mode (group format), duration (60 – 90 minutes per session), intervention goals (related to nutrition and physical activity), and the number of group leaders (two per group). Importantly, the intervention arms differ in how information is conveyed to parents, and how parents work towards attempting, achieving, and maintaining healthy cognitive and behavioural changes. All families are encouraged to manually monitor their daily nutrition, moderate-to-vigorous-physical activity (MVPA), sleep and leisure-time screen time (LTST) throughout the intervention period, and tracking tools are available for all family members (parents, children, other family members). To encourage monitoring, small prizes (e.g., cookbooks, activity-promoting toys and games) are awarded at three points during the 16-session intervention period (e.g., on week 6 for tracking completed in weeks 1 – 5, on week 11 for tracking completed in weeks 6 – 10, and on week 16 for tracking completed in weeks 11 – 15). Families are eligible to receive prizes in each of the three time periods regardless of their consistency with monitoring in previous time frames. For both CBT and PEP, families strive to achieve the same lifestyle goals (please see Table 2), which are based on evidence-based recommendations [55-57].

**Table 2:** Lifestyle goals for the CBT and PEP Versions of the PAC intervention

	
**1. Diet**	Daily vegetable and fruit intake: ≥5 servings
	Daily grain product intake: ≥50% servings as whole grains
	Daily sugar-sweetened beverage intake: 0 servings
**2. Physical Activity**	Daily steps: ≥12,000 (girls); ≥15,000 (boys)
	Daily moderate-to-vigorous physical activity: ≥90 minutes
**3. Sedentary Activity**	Daily leisure time screen time: ≤90 minutes

Fundamental differences do exist between the CBT and PEP interventions. CBT is a theoretically-based therapy that focuses on the role that cognitive processes play in the maintenance of problem behaviours, mood states, and habits [58]. CBT also highlights the relationship between thoughts, feelings and actions, and utilizes techniques involving motivation, goal-setting, problem-solving, and knowledge/skill acquisition that can facilitate sustainable behaviour changes [59]. This intervention is designed to promote parental adoption of a more authoritative parenting style, which is exemplified by elements including rational decision-making, verbal give-and-take, autonomous self-will, and disciplined conformity. CBT can improve parenting style and parent–child interactions [60], so group leaders work with parents who have more permissive (e.g., makes few demands) or authoritarian (e.g., restricts child autonomy) parenting approaches to help them to develop authoritative parenting skills. The skills learned in CBT help parents to identify and change the parenting mechanisms that maintain their children’s current lifestyle habits; group leaders help parents to link knowledge, attitudes, thoughts, and feelings to behaviours and facilitate setting incremental goals that build on one another week-to-week in a manner that corresponds to parent motivation. CBT encourages consistent participation and collaboration between group leaders and participants; a Socratic questioning approach is also applied to help parents find their own answers to problems instead of having questions answered or problem-solved by group leaders [61]. To ensure the CBT-based version adhered to the main tenets of cognitive behavioural interventions, we consulted an international, three-member panel of clinical and research experts in CBT, obesity, and intervention development and evaluation. Their critical evaluation, feedback, and recommendations for revisions were incorporated into the curriculum.

In contrast, PEP is a knowledge-based intervention that is modeled after traditional nutrition and health education programs. Information regarding healthier parenting styles is provided in a didactic manner without directive leader-initiated goal-setting or problem-solving. In relation to CBT, PEP is a more passive intervention and there is limited focus on active skill building. The intervention is predicated on a more traditional approach that assumes behaviour change results from increased knowledge, although different activities are included for parents that encourage learning (individual activities, brain-storming, hands-on experiential learning) [62]. While group discussions and problem-solving are included within PEP, active integration of learned concepts in goal-setting and linking cognitions and behaviours to lifestyle changes are not integral. There is no active cognitive or behavioural skill building process embedded in the program. In relation to CBT, PEP requires less investment of energy and participation from parents, which many parents prefer. While PEP does not represent a true control group, its content and delivery are consistent with what many clinicians provide for weight management.

### Intervention fidelity

One of the challenges in evaluating the effectiveness of lifestyle and behavioural interventions is optimizing the fidelity with which the interventions are delivered. In other words: During the trial, are the interventions delivered in the way in which they are intended? We are using multiple strategies to ensure standardization and consistency across parent/child cohorts as well as within and between intervention arms. Group leaders receive intervention delivery training through the use of standardized intervention manuals and weekly clinical meetings; following a manual and receiving reinforcement through team meetings ensures the interventions are delivered in a standard and consistent manner. Manualization of interventions is used extensively in health fields [63-66] and adds methodological rigor to our study design. Evaluation of delivery (adherence to intervention manuals) is achieved by session videotape. Three sessions within each of the CBT and PEP intervention arms are randomly chosen for videotaping and subsequently scored for adherence by two independent evaluators using a template created for the study that highlights study content and unique features of the interventions (See Table 3 for intervention adherence checklist). Inter-rater reliability is calculated using the Kappa statistic [67]. Issues with non-adherence to intervention integrity are addressed during weekly meetings.

**Table 3:** Intervention integrity checklist for the PAC intervention

Group Leader #1: __________________________________			Date (dd/mm/yyyy): ________________	
Group Leader #2: __________________________________			Rater: ___________________________	
**Content** – Are the following content items addressed in the session?			**Yes**	**No**
(Note: Details to be included by investigators; content items vary session-to-session)				
__________________________________________________________________________________________________________________				
__________________________________________________________________________________________________________________				
__________________________________________________________________________________________________________________				
__________________________________________________________________________________________________________________				
__________________________________________________________________________________________________________________				
**Content** – Did leaders follow the manual content throughout the session?				
(Rater: Please read along in the manual during session to evaluate adherence to content).				
Additional comments:				
___________________________________________________________________________________				
___________________________________________________________________________________				
___________________________________________________________________________________				
___________________________________________________________________________________				
___________________________________________________________________________________				
		
**Group Process Foundations**	**Leader 1**		**Leader 2**	
	**Yes**	**No**	**Yes**	**No**
Leader linked parent experiences to individual factors (e.g., linked the impact of parent, child preferences on experiences).				
Leader linked parent experiences to family factors (e.g., linked the impact of family members on parent experiences).				
Leader linked parent experiences to environmental factors (e.g., linked the role of community and available resources on parent experiences).				
Leader made links between thoughts, feelings and behaviours.				
Leader reflected questions back to group.				
Leader answered questions directly.				
Leader shared personal anecdotes/stories in response to parents sharing their experiences.				

### Outcome measures

Child BMI z-score is the primary outcome for this study. Several investigations [68-72] have included BMI z-score as the primary outcome in pediatric weight management studies since it represents a clinically-meaningful indicator of weight status in childhood. Beneficial changes in this variable are linked to improvements in obesity-related risk factors for chronic diseases including insulin resistance, dyslipidemia and high blood pressure [73,74]. The inclusion of this outcome is also informative when comparing effect sizes across studies.

Consistent with recommendations for evaluating the effects of pediatric obesity treatment [12,17], we are measuring a number of secondary physiological, behavioural, and psychosocial variables in families. Along with assessing nutrition and physical activity behaviours, anthropometry, and cardiometabolic risk factors, we are testing potential moderating (e.g., sex, age) and mediating (e.g., parents’ lifestyle behaviors) variables to explore how different child and parent features interact with and respond to the different interventions and hypothesize how they contribute to changes in child health outcomes [75]. This is a valuable, but often overlooked process that provides a better understanding of the mechanisms of lifestyle change. This approach, in turn, allows our team to improve our interventions since we can distinguish between effective and ineffective intervention components [76].

#### Physical exam, medical history, and demography

Physical exams (for children) and a family medical history interview (for parents) are conducted by a pediatric endocrinologist at clinic intake. In addition, children’s physical development are determined according to established guidelines [77,78]. Demographic data (e.g., date of birth, ethnicity, parents’ education, household income) are also collected.

#### Anthropometry

Anthropometric measurements for children and parents are collected using a standardized protocol. Height is measured (without shoes) to the nearest 0.1 cm using a Seca 242 wall-mounted electronic stadiometer (Seca, Hamburg, Germany). Weight is assessed (wearing light clothing) to the nearest 0.1 kg using a Seca 644 medical scale. BMI is then calculated. For children, BMI data are entered into *EpiInfo* (Center of Disease Control and Prevention, Atlanta, GA) to determine sex- and age-specific BMI z-scores and percentiles. Waist circumference (WC) is measured at the top of the iliac crest using a spring-loaded Gulick anthropometric tape (FitSystems Inc., Calgary, AB). WC data are acquired using the following protocol: Measurements are performed with the tape measure snugly positioned on, but not compressing, the skin; the spring is pulled until calibration tension is achieved on the tape. Clothing is positioned so the abdomen is exposed (bottom of shirt is positioned below the arms, which are crossed over the chest; pants/shorts are loosened and lowered slightly to reveal the hips). Recordings are taken at the end of a normal expiration and not during breath holding or abdominal muscle contractions. This procedure is repeated, and if the first and second values differ by more than 0.5 cm, a third measurement is taken. Values are then averaged and recorded to the nearest 0.1 cm.

#### Dietary intake

Dietary intake is recorded prospectively by children and parents over four consecutive days, and includes one weekend day. Once records are returned by families, a registered dietitian (RD) reviews all nutrition information with families to clarify items, validate portion sizes and name brands, and to ensure data completeness. Subsequently, these data are entered into the *Food Processor Diet Analysis* software program (ESHA Research, Salem, OR) to determine number of servings from *Eating Well with Canada’s Food Guide *[55], total energy, macro-nutrient, and dietary fiber intakes.

#### Physical activity and sedentary behaviours

Children and parents measure prospectively their total physical activity using a pedometer (Yamax Digi-Walker SW-200) over a 7-day interval that overlaps with their dietary intake recording. Families receive training on proper pedometer positioning, data recording, and other physical activity monitoring details before the measurement interval starts. To ensure proper functioning, all pedometers are calibrated prior to use by way of a standardized step count test. Since pedometers do not capture information regarding physical activity intensity, children and parents complete simultaneously a 7-day physical activity record to document quality, quantity, and patterns of moderate-to-vigorous physical activity, leisure-time screen time (television, computer, video game, etc.), and sleep duration. Once records are returned, data are reviewed with families to ensure completeness and clarify durations and intensities of activities.

#### Fasting blood sample

A fasting blood sample (~3 ml) is retrieved from children to assess total cholesterol, HDL-cholesterol, LDL-cholesterol, triglyceride, insulin, and glucose levels. Using insulin and glucose values, the homeostatic model of insulin resistance (HOMA-IR) is calculated [79]. Blood collections may occur at a number of different community-based laboratories in the Edmonton-area; however, all analyses are completed centrally at the University of Alberta Hospital laboratory to ensure the same analytical procedures are used throughout the duration of this trial.

#### Blood pressure

With children in the seated position (and following a five minute rest), systolic and diastolic blood pressure (SBP and DBP, respectively) are measured oscillometrically with an appropriately-sized cuff on the right arm. SBP and DBP are measured three times with one minute intervals between each assessment. Representative values are based on the average of the three measures.

#### Questionnaires

Children complete the *Piers-Harris Children’s Self-Concept Scale (2*^*nd*^*edition) *[80] to derive an assessment of their self-concept that focuses on the self-perceptions children have of themselves and their behaviors related to others, the *Child Depression Inventory *[81] to assess depressive symptomatology, and the *Pediatric Health-Related Quality of Life (PedsQL 4.0)* to assess functioning related to physical, emotional, social and scholastic domains [82,83]. Parents complete the *Parenting Stress Index*, which assesses three subscales reflecting child and parent relationship characteristics and life stressor [84], the *Child Behaviour Checklist *[85], which probes child behavior and potential psychopathology, the *Family Adaptability and Cohesion Evaluation Scales IV,* which assesses family cohesion and flexibility dimensions, family communication and satisfaction [86], the *Lifestyle Behaviors Checklist *[87,88] to survey common parental concerns regarding children's eating behaviors, physical activity, and other weight-related behaviors, and the parent proxy report of the *PedsQL 4.0*.

#### Family enrolment, intervention dose, and attrition

**Figure 1:**
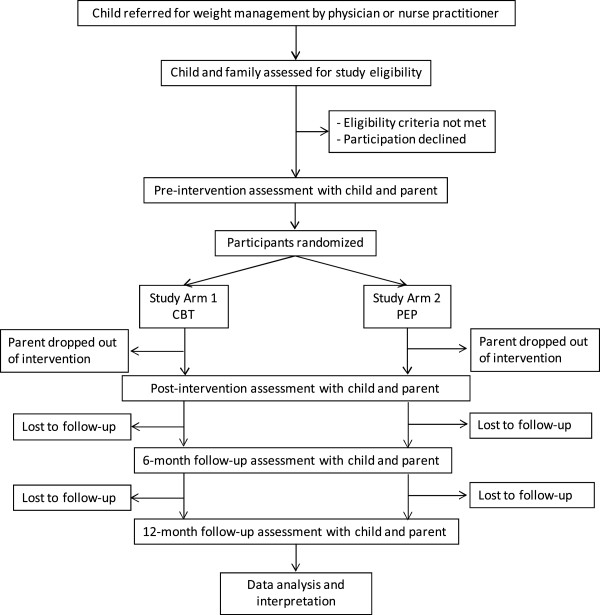
Participant flow diagram according to the CONSORT statement.

The study research coordinator record several study-specific variables that will be used to populate the CONSORT diagram (Figure 1). For instance, the total number of families recruited and enrolled in the study, parent attendance at each of the PAC sessions, and time of and reason for dropping out of the study are all documented.

### Sample size

We are enrolling 90 families (n = 45 families × 2 PAC intervention arms) in this study. Based on our clinical experience, we anticipate a 33% level of attrition, which should yield post-intervention data on 60 families. With a total sample size of 60 families (n = 30 per arm), we are able to detect a minimum difference of 0.5 in BMI z-score change between the CBT and PEP intervention groups. To our knowledge, no experimental work has been done to define specific weight-related goals for pediatric obesity interventions. However, our 0.5 BMI z-score difference is based on a review by Epstein and colleagues that suggested a reduction of this magnitude is clinically meaningful [89]. Sample size calculations were completed using *PASS 2008* (http://www.ncss.com), and assumed a standard deviation (SD) of 0.83 for each group, a one-sided significance level of 0.05, and a desired power of 80%. This SD was based on preliminary findings that included pre- and post-intervention BMI z-score data [90]. Multiple linear regressions are able to detect an R^2^ value of 10% for the effect of intervention on percent change in BMI z-score at a 0.05 significance level with 80% power, assuming that the inclusion of five control variables explains 20% of the variability in change in BMI z-score.

### Randomization

Parents are randomly assigned to either the CBT-based or PEP-based PAC intervention group following their completion of pre-intervention assessments, but before the initiation of the PAC sessions. Given that parents and their children are recruited and enrolled into the group-based interventions in several cohorts, we use a computer-generated, block randomization approach, which is completed by one of our research team members who has no direct contact with participants. Parents will be blinded to group assignment, and while they are aware that there are two groups of parents receiving the PAC intervention, they are unaware of the modality and methodological differences between CBT and PEP.

### Statistical analysis

Data cleaning, analyses, figures, tables, and statistical sections for dissemination are conducted by a blinded biostatistician with the Biostatistics Consulting Group within the Women and Children’s Health Research Institute (http://www.wchri.org) at the University of Alberta. Spotfire S + 8.2 (TIBCO Software Inc, 2010) and SAS 9.3 (SAS Institute Inc, 2010) will be used for analyses. Analyses are conducted both with PAC intervention completers only and with all participants (to evaluate effectiveness). Continuous variables are described by summaries (e.g., means, medians, ranges and SDs) and frequency distributions are determined for categorical variables. For the primary and secondary outcome variables, the percent change from pre- to post-intervention (as well as 6- and 12-months follow-up) for both groups is determined and summarized along with the 95% confidence intervals (CIs) for mean percent change. Boxplots and histograms are used to display the percent change for these variables; bar charts are used for categorical variables. Our analyses are conducted separately for each mean percent change at each observation time (i.e., pre-intervention to post-intervention, pre-intervention to 6-month follow-up, and pre-intervention to 12-month follow-up). Statistical tests are set at conventional levels (p < 0.05) and all analyses are intention-to-treat. Multiple imputation methods may be used in the event of missing data. To minimize the risk of bias, a research team member with no direct contact with enrolled families is leading the data analyses.

To compare interventions, one-sided independent samples Student’s t-test assesses percent changes in BMI z-score (primary objective); if required, data transformations are used to ensure data normality. A similar process is used to determine intervention-mediated changes in other physiological, behavioural, and psychosocial outcomes (secondary objectives). In addition, the percentage of families with improved categorical outcomes (i.e., those who do/do not meet recommendations from *Eating Healthy with Canada’s Food Guide*) is compared across interventions using a one-sided independent samples proportion tests. A multivariable linear regression model is developed for the primary outcome (percent change in BMI z-score) to determine the intervention group effect while controlling for potential confounders (i.e., cohort, percent attendance, household income). If any of the intervention groups include siblings, CIs are adjusted using variance inflation factors, and univariable or multivariable random intercept mixed effects models are used in lieu of Student’s t-tests and multivariable linear regression, respectively. Analogous calculations are made to compare the percent change from pre-intervention to 12-month follow-up, although intervention attrition may reduce our power to detect a significant difference. Mixed effect models are developed to examine the effects of the interventions on primary and secondary outcomes over time. This approach allows all pre-intervention, post-intervention, and follow-up data to be included in our modeling. For all models, residual diagnostics are used to assess model assumptions and appropriate transformations and/or non-linear terms are included as necessary. Our reporting of study results is in accordance with the CONSORT statement [91].

## Discussion

With overweight and obesity impacting the lives of at least two million boys and girls (and their families) in Canada [2,92], there is a clear need for effective interventions to promote successful pediatric weight management. This RCT builds on a solid evidence base of family-based interventions for managing pediatric obesity [89] as well as cognitive behavioral strategies to facilitate healthy changes in cognitions and lifestyle behaviors [59]. Contemporary research has demonstrated clearly that PAC interventions that are designed to manage pediatric health issues can lead to positive outcomes for both children and families [36-38], which includes a growing body of literature in the area of pediatric weight management [40-44]. This totality of evidence led us to hypothesize that children with obesity whose parents complete the CBT-based PAC intervention will achieve greater reductions in adiposity, improved lifestyle behaviours and psychosocial outcomes, and decreased cardiometabolic risk factors compared to children whose parents complete the PEP-based PAC intervention.

### Strengths and limitations

The current study has a number of noteworthy strengths. First, we took a rigorous and systematic approach to the design of this RCT as well as the development and refinement our intervention materials for parents and group leaders. The manualized intervention materials for group leaders, external expert review of intervention materials, and plan to evaluate intervention fidelity enhance methodological rigour and provide confidence that the interventions are distinct from one another, which is needed to evaluate their differential effects. Second, if the CBT and/or PEP versions of the PAC intervention help parents to make positive changes, the manualization of intervention materials enables the dissemination and evaluation of PAC in other settings (i.e., primary care, public health). Existing partnerships between our team members and health services (e.g., Alberta Health Services; http://www.albertahealthservices.ca) and non-governmental (e.g., Canadian Obesity Network; http://www.obesitynetwork.ca) organizations should facilitate the dissemination and future delivery of the PAC intervention within the province of Alberta and across Canada. Third, because most of the boys and girls enrolled in this study are recruited from the PCWH, they are almost exclusively described as *severely obese* (BMI ≥99^th^ percentile or BMI ≥40 kg/m^2^) [93]. Since much of the evidence in managing pediatric obesity includes less overweight and obese participants [89], the present RCT contributes important information regarding the impact of parent-based approaches for managing severe obesity in children. Finally, offering pediatric weight management interventions for parents exclusively represents an efficient model of care. Common sense dictates that the PAC interventions can be delivered using fewer resources (physical, human, financial) than interventions that include both parents and children. Since many pediatric weight management centers in Canada struggle to deliver health services with limited resources and infrastructure [14], interventions that are both efficient and effective are most desirable.

It is also important to acknowledge the potential limitations of this study. First, while we are implementing several retention strategies (e.g., regular telephone calls to confirm appointment and session attendance, modest incentives for families to complete outcome assessments at study time points), we know from our own clinical and research experience [48] and the published literature [94] that lack of engagement and attrition are common phenomena in pediatric weight management. These issues may have a negative impact on family recruitment and retainment within this RCT. To better understand and potentially address issues of engagement and attrition, we are currently completing an independent multi-center, qualitative study to interview 100 parents and 100 children and youth with obesity in order to understand parents’ and children’s decisions regarding engagement in and attrition from pediatric weight management centers [95]. These emerging data should help to inform decisions that should optimize recruitment and retention in the current study. Second, there is an increasing number and variety of services that relate to pediatric weight management (i.e., physical activity and exercise programs for families, community-based weight management programs, and outpatient nutrition) available to families in the Edmonton-area. Although this is advantageous from family and health services viewpoints, this reality decreases the number of local families who may be enrolled in our study. Third, our study is conducted in an outpatient clinic setting, so the health professionals performing many of the outcome assessments (e.g., anthropometry) know which families were randomized to the CBT and PEP intervention groups. Although they have no vested interest in the study as study co-investigators, this real-world environment introduces a potential source of bias (lack of assessor blinding; [52]) because all families receiving weight management care are regularly discussed during weekly case conference team meetings. Finally, to date, our discussions with families regarding a PAC approach for managing pediatric obesity have occasionally been met with resistance; since children present with the identified health concern, parents are sometimes taken by surprise with the primary role they are asked to play in the PAC intervention. Many parents embrace their leadership role in making and sustaining healthy changes within their families, although other parents will prefer that intervention efforts focus on children themselves or include both parents and children. As a consequence of these differing perspectives and intervention foci, some parents decline study participation.

The current study provides a unique opportunity by combining CBT and PAC within an intervention model to improve the health and well-being of children with obesity and their parents in a real-world, outpatient clinical setting. Findings from this research should contribute important insight into how health services can be provided effectively and efficiently to families in order to address pediatric obesity.

## Competing interests

In relation to the present research, all authors are in agreement that they have no financial or non-financial interests to declare.

## Authors’ contributions

GDCB conceived of the study (with ASN), co-authored a first draft of the manuscript (with KAA), and co-led the development of the PAC interventions (with RAK and ASN). KAA co-authored a first draft of the manuscript (with GDCB). RAK co-led the development of the PAC interventions (with GDCB and ASN) and assisted with study design as well as writing and editing the manuscript. RJR authored the sample size and statistical analysis sections of the manuscript and assisted with study design as well as writing and editing the manuscript. NLH assisted with study design as well as writing and editing the manuscript. JCS assisted with study design as well as writing and editing the manuscript. MMJ assisted with study design as well as writing and editing the manuscript. AMS assisted with study design as well as writing and editing the manuscript. ASN conceived of the study (with GDCB), co-led the development of the PAC interventions (with GDCB and RAK), and assisted with writing and editing the manuscript. All authors have read and approved the final manuscript.

## Pre-publication history

The pre-publication history for this paper can be accessed here:

http://www.biomedcentral.com/1471-2431/12/114/prepub

## Supplementary Material

Additional file 1 **Appendix 1. **Sample screenshot from the PAC Intervention Leader Manual, which includes the PowerPoint ® slide presented to parents, bullet points for group leaders to emphasize/paraphrase with parents, and references used to inform the development of the evidence-based curriculum. **Appendix 2. **Sample screenshot from the PAC Intervention Parent Manual, which includes the PowerPoint ® slide presented to parents, space for parents to record the results of their goal-setting from the previous week, and probing questions to encourage parents to explore their thoughts, feelings and behaviours, and what (if anything) they would do differently next time. Click here for file
