# Supplementary material for: Parents as Agents of Change (PAC) in pediatric weight management: The protocol for the PAC randomized clinical trial
Source: BMC Pediatr. 2012 Aug 6;12:114. doi: 10.1186/1471-2431-12-114 (PMC3469386; doi:10.1186/1471-2431-12-114)
Supplement: Additional file 1 — Appendix 1. Sample screenshot from the PAC Intervention Leader Manual, which includes the PowerPoint ® slide presented to parents, bullet points for group leaders to emphasize/paraphrase with parents, and references used to inform the development of the evidence-based curriculum. Appendix 2. Sample screenshot from the PAC Intervention Parent Manual, which includes the PowerPoint ® slide presented to parents, space for parents to record the results of their goal-setting from the previous week, and probing questions to encourage parents to explore their thoughts, feelings and behaviours, and what (if anything) they would do differently next time. [file 1471-2431-12-114-S1.doc]

**Appendix 1. Sample screenshot from the PAC Intervention Leader Manual, which includes the PowerPoint ® slide presented to parents, bullet points for group leaders to emphasize/paraphrase with parents, and references used to inform the development of the evidence-based curriculum.**

**Appendix 2. Sample screenshot from the PAC Intervention Parent Manual, which includes the PowerPoint ® slide presented to parents, space for parents to record the results of their goal-setting from the previous week, and probing questions to encourage parents to explore their thoughts, feelings and behaviours, and what (if anything) they would do differently next time.**
